# Supplementary material for: Compositional heterogeneity near the base of the mantle transition zone beneath Hawaii
Source: Nat Commun. 2018 Mar 28;9:1266. doi: 10.1038/s41467-018-03654-6 (PMC5872023; doi:10.1038/s41467-018-03654-6)
Supplement: Supplementary file 1 — Supplementary Information (PDF 5173 kb) [file 41467_2018_3654_MOESM1_ESM.pdf]

**Supplementary Information**

# Compositional heterogeneity near the base of the mantle transition zone beneath Hawaii

Chunquan Yu[1, 2*], Elizabeth A. Day[1,3], Maarten V. de Hoop[4], Michel Campillo[1,5], Saskia Goes[3], Rachel A. Blythe[3], Robert D. van der Hilst[1]

[1] *Department of Earth, Atmospheric, and Planetary Sciences, Massachusetts Institute of Technology, Cambridge, MA 02139, USA*

[2] *Now at Seismological Laboratory, California Institute of Technology, Pasadena, CA 91125, USA*

[3] *Department of Earth Science and Engineering, Imperial College, London, SW7 2BP, UK*

[4] *Department of Computational & Applied Mathematics, Rice University, Houston, Texas 77005, USA*

[5] *Institut des Sciences de la Terre, Université Joseph Fourier, BP 53X, 38041 Grenoble, France*

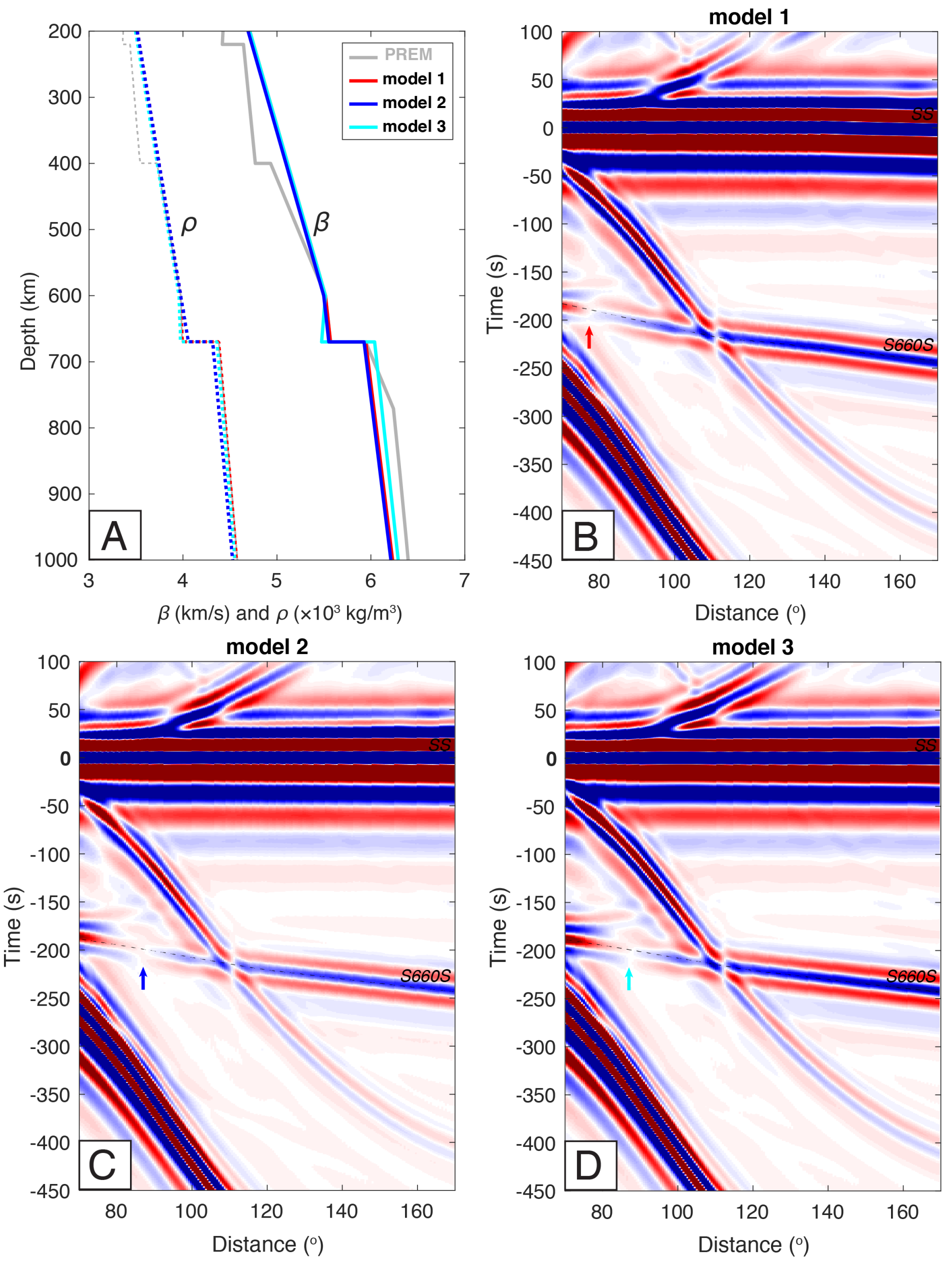


Supplementary Figure 1 Synthetic waveform modeling of three different models. (A) Density and $V_S$ profiles. All models are modified from PREM [1] with only one sharp discontinuity at *660*. (B), (C) and (D) are synthetic waveforms from models 1, 2, and 3, respectively. Red, green and blue arrows mark the zero-reflection distance across which the polarity of $S^{660}S$ changes.

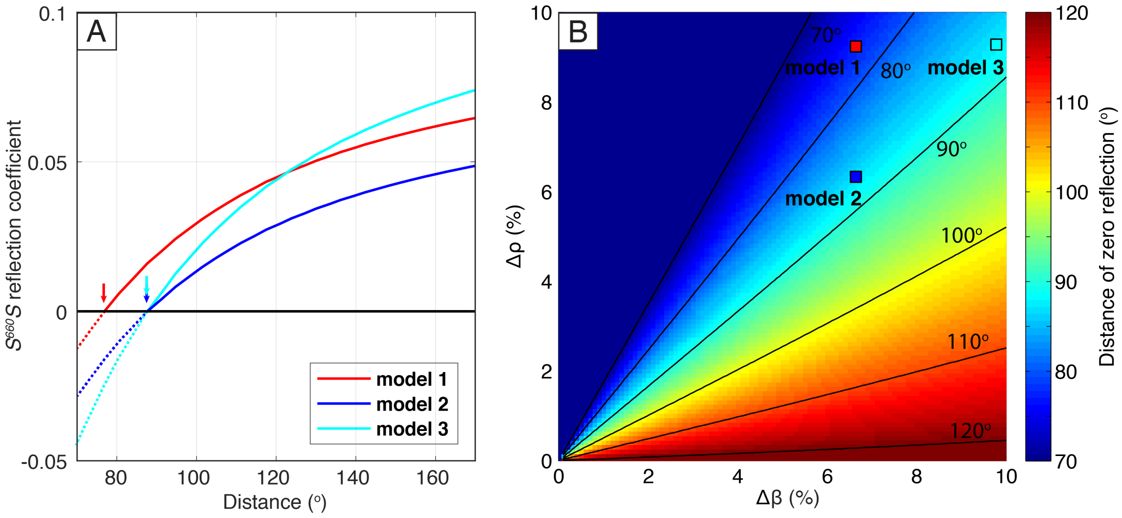


Supplementary Figure 2 Relationships between $S^{660}S$ reflection coefficient, source-receiver (or epicentral) distance, and contrasts in wave speed and density ($\Delta\beta$, $\Delta\rho$). (A) $S^{660}S$ reflection coefficients as a function of distance calculated for three different combinations of $\Delta\beta$ and $\Delta\rho$. Zero-reflection distances are consistent with synthetic waveforms produced from these models (Supplementary Fig. 1). (B) Distance of zero reflection as a function of $\Delta\beta$ and $\Delta\rho$ across *660*. Note that for fixed zero-reflection distance, the ratio between $\Delta\beta$ and $\Delta\rho$ is almost constant (as it is for models 2 and 3). Mean values for $\Delta\beta$ and $\Delta\rho$ at *660* are from PREM.

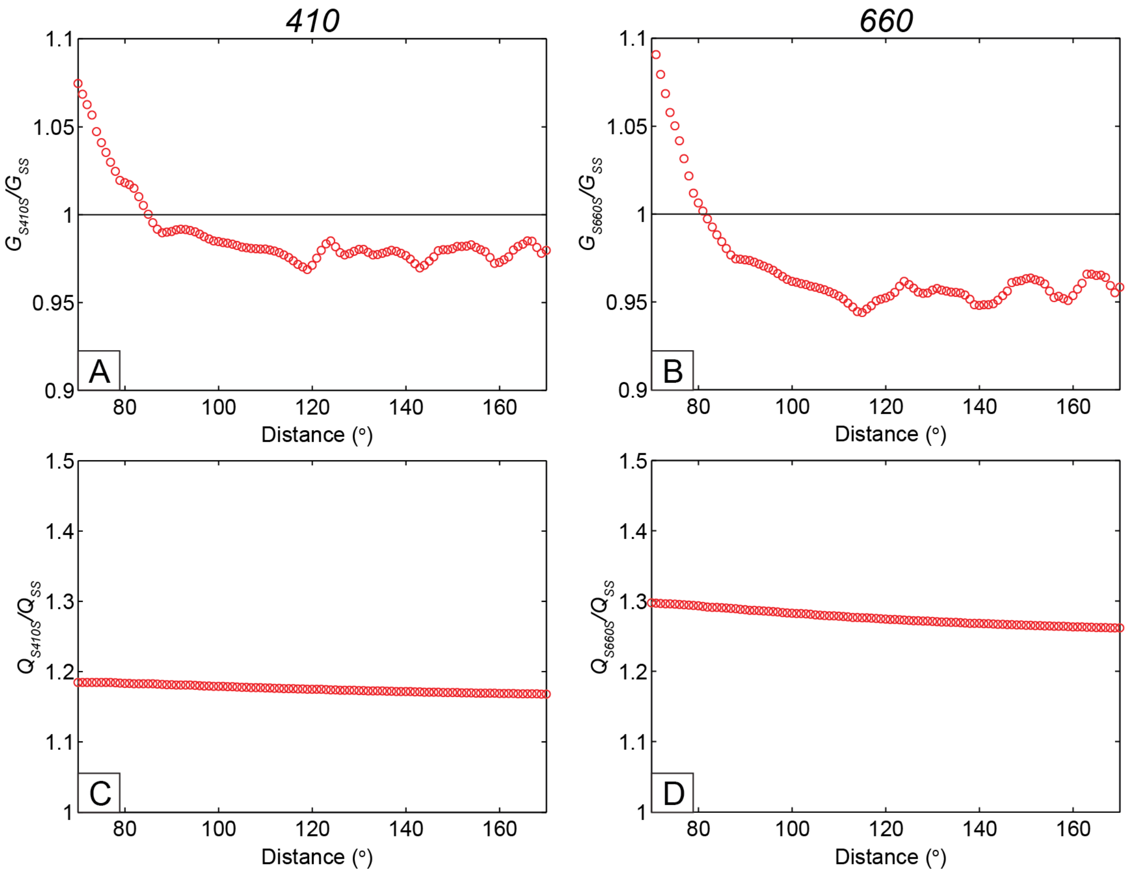


Supplementary Figure 3 Relative geometrical spreading (G) and relative quality factor (Q) between *SS* precursors and the reference *SS*. (A) and (B) are $G_{S410S}/G_{SS}$ and $G_{S660S}/G_{SS}$, respectively. (C) and (D) are $Q_{S410S}/Q_{SS}$ and $Q_{S660S}/Q_{SS}$, respectively. The quality factor (or attenuation) correction is calculated for a central frequency of 1/30 Hz using the PREM attenuation model.

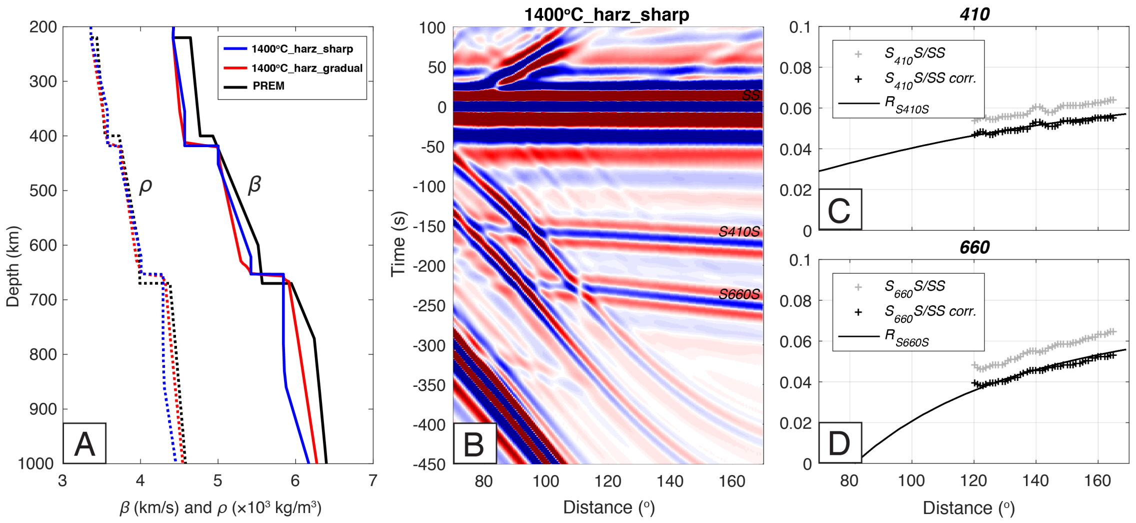


Supplementary Figure 4 Synthetic verification of $S^dS/SS$ amplitude ratios to $S^dS$ reflection coefficients conversion using eqn. (4). (A) Three different 1D models. (B) Synthetic waveforms for model "1400°C_harz_sharp" (green lines in A). (C) Comparison between theoretical $S^{410}S$ reflection coefficient (black line) and that converted from observed $S^{410}S/SS$ amplitude ratio after corrections for geometrical spreading and attenuation (black plus). (D) Same as (C) but for *660*.

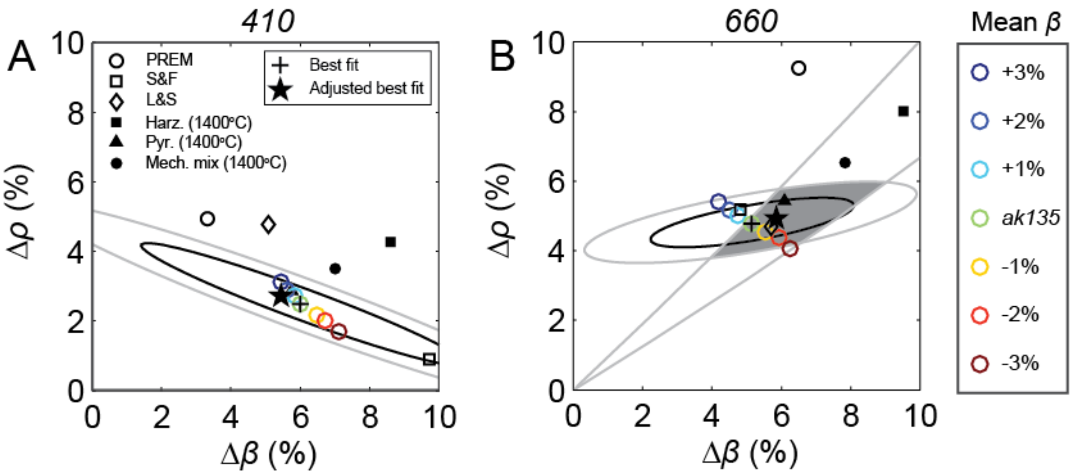


Supplementary Figure 5 Estimated density and $V_S$ contrasts across the (A) *410* and (B) *660* for the entire study region. Black and gray ellipses mark the 1-sigma and 2-sigma limits of parameters in the model space. Light shaded region in (B) marks the additional 2-sigma limits of parameters from estimates of zero reflection distance (93±5°; Fig. 2D). Black star marks our best fitting model. Color-coded circles show the trade-off of parameters with mean $V_S$ across the discontinuity. The PREM model is marked as a black open circle. S&F represents the global model of *Shearer and Flanagan* [2]. L&F represents the global model of *Lawrence and Shearer* [3]. Also shown are estimates from thermodynamic modeling for mantle composition of harzburgite and pyrolite at an adiabatic temperature of 1400 °C.

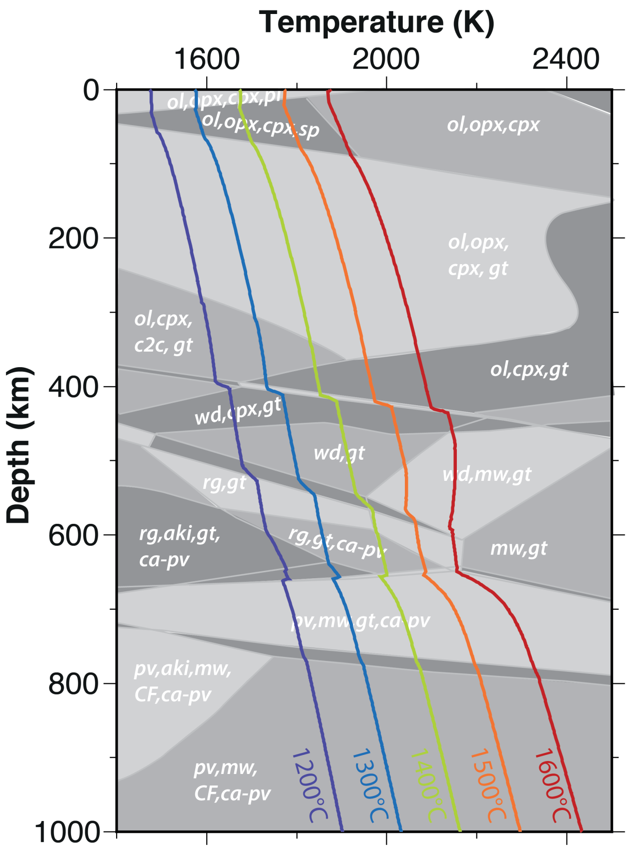


Supplementary Figure 6 Main phase boundaries in a pyrolite (composition from Xu et al. [4]) in gray, computed with Perple_X [5] and the database from Stixrude and Lithgow-Bertelloni [6], with the different adiabats superimposed (potential temperatures between 1200 °C and 1600 °C) that are used for the velocity computations. Abbreviations: ol – olivine, opx – orthopyroxene, cpx – clinopyroxene, gt – garnet, c2c – high-pressure clinopyroxene, wd – wadsleyite, mw – magnesiowüstite, rg – ringwoodite, aki-akimotoite, CF – calcium-ferrite, ca-pv – calcium-perovskite, pv – bridgmanite.

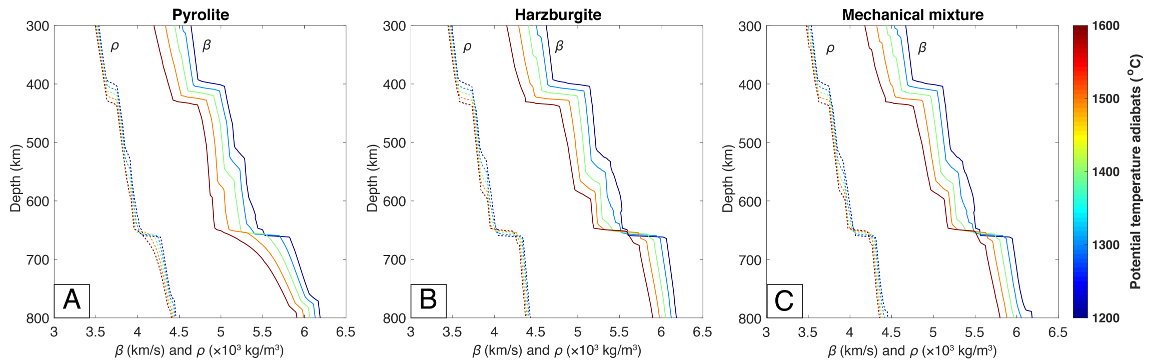


Supplementary Figure 7 Synthetic density and $V_S$ profiles for (A) pyrolite, (B) harzburgite and (C) mechanical mixture mantle compositions. Color-coded lines show the effect of mantle temperature on modeling results.

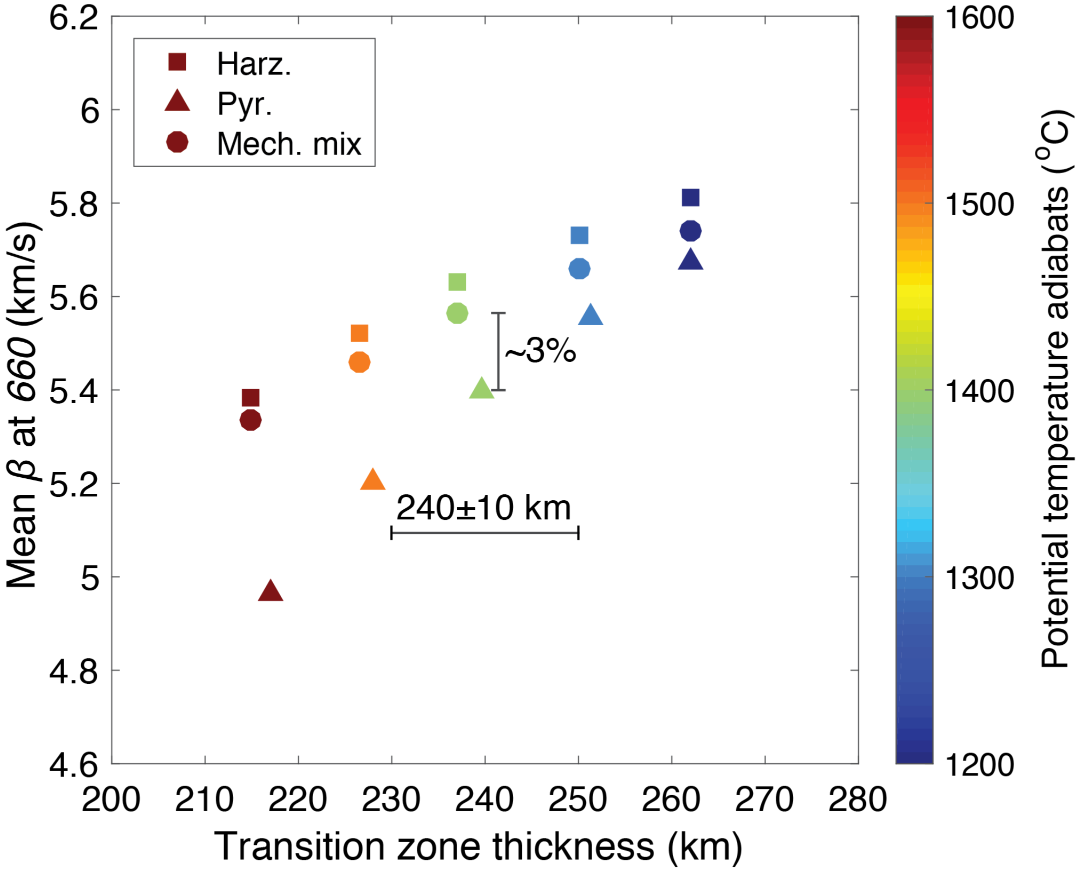


Supplementary Figure 8 Mean shear wave speed at *660* versus the mantle transition zone thickness from thermodynamic modeling for three different mantle compositions at various thermal conditions. Note that the transition zone thickness is most sensitive to temperature, but the mean shear wave speed is sensitive to both temperature and composition. The depths of *410* and *660* from synthetic profiles (Supplementary Fig. 7) are set at where the $V_S$ or density gradient is largest.

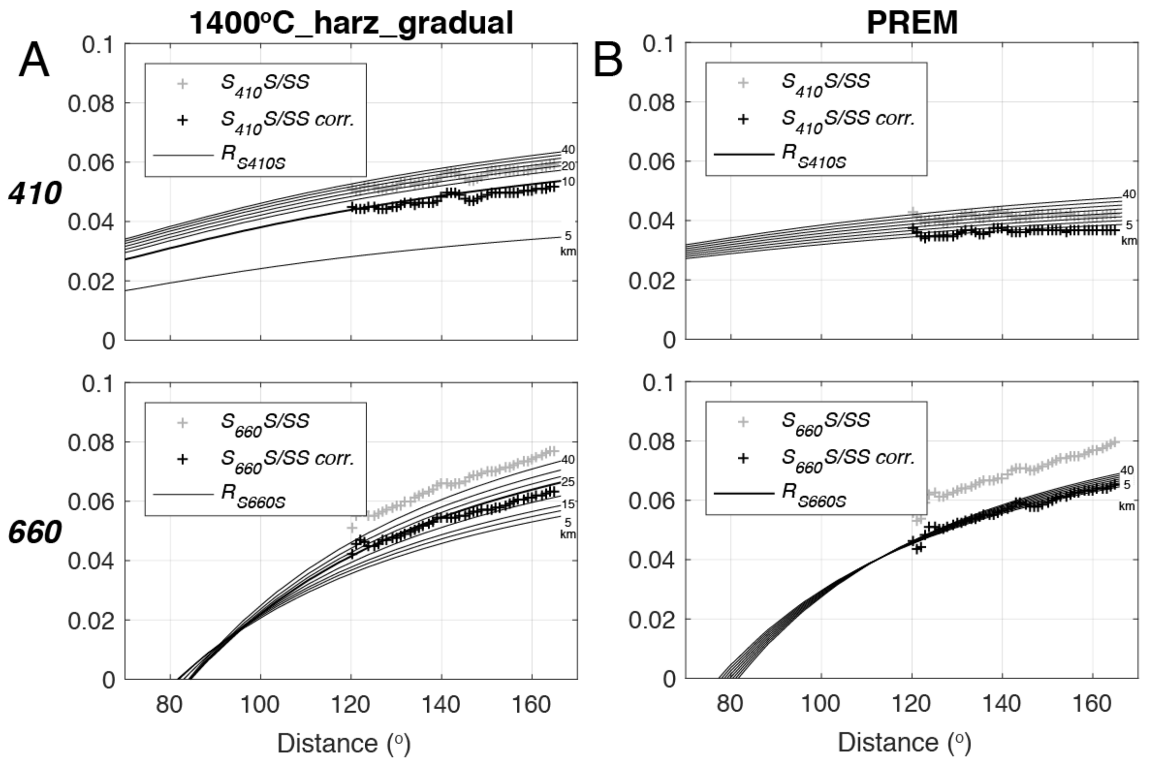


Supplementary Figure 9 Estimation of an equivalent depth interval for reflection coefficient calculation. (A) and (B) are for models "1400ºC_harz_gradual" and PREM, respectively (Supplementary Fig. 4A). Reflection coefficients are calculated based on the total density and $V_S$ contrasts over the depth interval (centered at where the $V_S$ or density gradient is largest) through eqn. (4). $S^{410}S/SS$ and $S^{660}S/SS$ are corrected for geometrical spreading and attenuation.

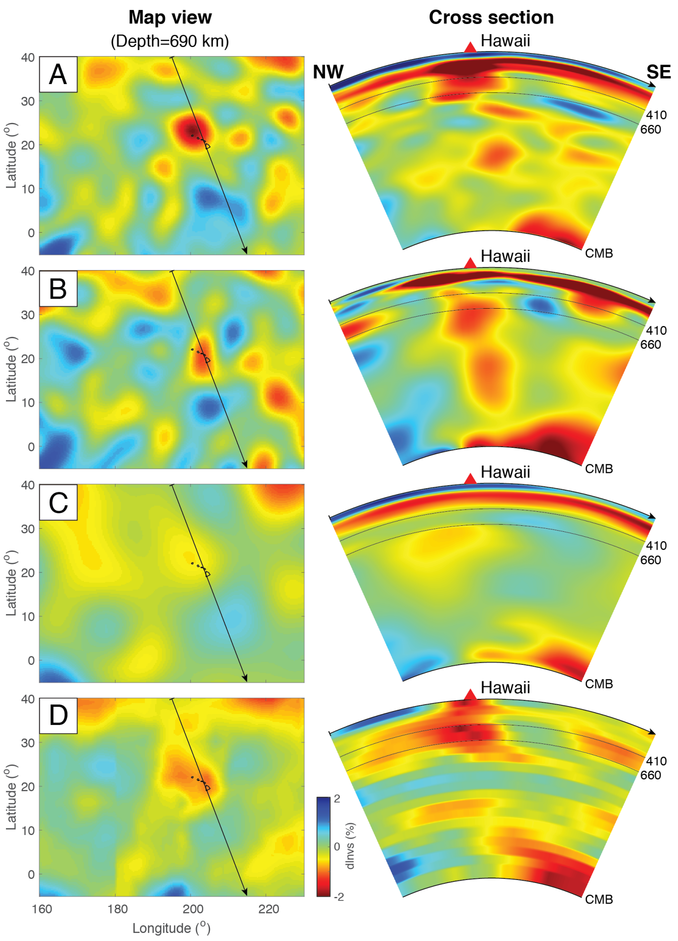


Supplementary Figure 10 3D variation in shear wave speed in the mantle beneath the Hawaii region according to the tomographic models used by *Yu et al.* [7] to correct *SS* travel times for topography measurement. (A) S40RTS [8], (B) SEMUCB-WM1 [9], (C) S362WMANI [10], and (D) GyPSuM [11]. The left and right columns show $V_S$ perturbation in the uppermost lower mantle (depth=690 km) and along a NW-SE profile, respectively. The high wave speed anomalies just below *660* that are visible SE of Hawaii (most clearly in S40RTS) could be due to the local harzburgite enrichment discovered and quantified with the reflectivity analysis presented in this study.

**Supplementary References**


1. Dziewonski AM, Anderson DL. Preliminary reference Earth model. *Phys Earth Planet Inter* **25**, 297-356 (1981).

2. Shearer PM, Flanagan MP. Seismic velocity and density jumps across the 410- and 660-kilometer discontinuities. *Science* **285**, 1545-1548 (1999).

3. Lawrence JF, Shearer PM. Constraining seismic velocity and density for the mantle transition zone with reflected and transmitted waveforms. *Geochem Geophys Geosyst* **7**, 10012 (2006).

4. Xu W, Lithgow-Bertelloni C, Stixrude L, Ritsema J. The effect of bulk composition and temperature on mantle seismic structure. *Earth Planet Sci Lett* **275**, 70-79 (2008).

5. Connolly JAD. Computation of phase equilibria by linear programming: a tool for geodynamic modeling and its application to subduction zone decarbonation. *Earth Planet Sci Lett* **236**, 524-541 (2005).

6. Stixrude L, Lithgow-Bertelloni C. Thermodynamics of mantle minerals-II. Phase equilibria. *Geophys J Int* **184**, 1180-1213 (2011).

7. Yu C, Day EA, de Hoop MV, Campillo M, van der Hilst RD. Mapping mantle transition zone discontinuities beneath the Central Pacific with array processing of SS precursors. *J Geophys Res: Solid Earth* **122**, 2017JB014327 (2017).

8. Ritsema J, Deuss A, Van Heijst H, Woodhouse J. S40RTS: a degree-40 shear-velocity model for the mantle from new Rayleigh wave dispersion, teleseismic traveltime and normal-mode splitting function measurements. *Geophys J Int* **184**, 1223-1236 (2011).

9. French S, Romanowicz B. Whole-mantle radially anisotropic shear velocity structure from spectral-element waveform tomography. *Geophys J Int* **199**, 1303-1327 (2014).

10. Kustowski B, Ekström G, Dziewoński A. Anisotropic shear - wave velocity structure of the Earth's mantle: A global model. *J Geophys Res: Solid Earth* **113**, (2008).

11. Simmons NA, Forte AM, Boschi L, Grand SP. GyPSuM: A joint tomographic model of mantle density and seismic wave speeds. *J Geophys Res: Solid Earth* **115**, (2010).
